# cAMP at Perinuclear mAKAPα Signalosomes Is Regulated by Local Ca^2+^ Signaling in Primary Hippocampal Neurons

**DOI:** 10.1523/ENEURO.0298-20.2021

**Published:** 2021-02-23

**Authors:** Tomasz Boczek, Qian Yu, Ying Zhu, Kimberly L. Dodge-Kafka, Jeffrey L. Goldberg, Michael S. Kapiloff

**Affiliations:** 1 Department of Ophthalmology, Byers Eye Institute, Mary M. and Sash A. Spencer Center for Vision Research; 2 Department of Medicine and Stanford Cardiovascular Institute, Stanford University School of Medicine, Palo Alto, CA 94034; 3 Department of Molecular Neurochemistry, Medical University of Lodz, 92-215 Lodz, Poland; 4 Calhoun Center for Cardiology, University of Connecticut Health Center, Farmington, CT 06030

**Keywords:** AKAP, cAMP, compartment, FRET imaging, PKA, signaling

## Abstract

The second messenger cyclic adenosine monophosphate (cAMP) is important for the regulation of neuronal structure and function, including neurite extension. A perinuclear cAMP compartment organized by the scaffold protein muscle A-kinase anchoring protein α (mAKAPα/AKAP6α) is sufficient and necessary for axon growth by rat hippocampal neurons *in vitro*. Here, we report that cAMP at mAKAPα signalosomes is regulated by local Ca^2+^ signaling that mediates activity-dependent cAMP elevation within that compartment. Simultaneous Forster resonance energy transfer (FRET) imaging using the protein kinase A (PKA) activity reporter AKAR4 and intensiometric imaging using the RCaMP1h fluorescent Ca^2+^ sensor revealed that membrane depolarization by KCl selectively induced activation of perinuclear PKA activity. Activity-dependent perinuclear PKA activity was dependent on expression of the mAKAPα scaffold, while both perinuclear Ca^2+^ elevation and PKA activation were dependent on voltage-dependent L-type Ca^2+^ channel activity. Importantly, chelation of Ca^2+^ by a nuclear envelope-localized parvalbumin fusion protein inhibited both activity-induced perinuclear PKA activity and axon elongation. Together, this study provides evidence for a model in which a neuronal perinuclear cAMP compartment is locally regulated by activity-dependent Ca^2+^ influx, providing local control for the enhancement of neurite extension.

## Significance Statement

Cyclic adenosine monophosphate (cAMP)-dependent signaling has been implicated as a positive regulator of neurite outgrowth and axon regeneration. However, the mechanisms regulating cAMP signaling relevant to these processes remain largely unknown. Live cell imaging techniques are used to study the regulation by local Ca^2+^ signals of a muscle A-kinase anchoring protein α (mAKAPα)-associated cAMP compartment at the neuronal nuclear envelope, providing new mechanistic insight into CNS neuronal signaling transduction conferring axon outgrowth.

## Introduction

CNS neurons responsible for higher order functions fail to survive or regenerate their axons after injury, resulting in permanent disability in common diseases such as stroke, Alzheimer’s disease, Parkinson’s disease and glaucoma. To combat this disability, strategies are being sought to promote CNS neuron survival and axon regeneration after injury, including the identification of intracellular signaling pathways whose activation might be beneficial in disease. Enhanced cyclic adenosine monophosphate (cAMP) signaling has been shown to potentiate neurotrophic signaling and to promote neuron survival and axon regeneration (Wang et al., 2015; Wild and Dell'Acqua, 2018). cAMP associated with these processes can be activity dependent (Goldberg and Barres, 2000; Goldberg et al., 2002; Corredor et al., 2012), but the mechanisms conferring this regulation remain largely unknown.

Although cAMP is in theory a freely diffusible second messenger present throughout the cell, it is now established that the specific effects of cAMP signaling in response to different stimuli often occur in discrete intracellular compartments organized by scaffold proteins that form multimolecular signaling complexes or “signalosomes” (Wild and Dell'Acqua, 2018). Scaffolds that bind the cAMP effector protein kinase A (PKA) are called A-kinase anchoring proteins (AKAPs). Diverse neuronal functions, including synaptic plasticity, neuronal excitability and transduction of sensory information, have been shown to be associated with AKAP-mediated compartmentation (Wild and Dell'Acqua, 2018). Recent studies have implicated muscle AKAPα (mAKAPα) in prosurvival and progrowth neurotropic and cAMP signal transduction, including in the extension of neurites by hippocampal and retinal neurons *in vitro* (Wang et al., 2015; Boczek et al., 2019).

Expressed in neurons and striated myocytes, the large 250-kDa mAKAP (AKAP6) scaffold (α-isoform in neurons, β-isoform in myocytes) is localized to the nuclear envelope via binding to the Klarsicht/ANC-1/Syne-1 homology (KASH) domain, transmembrane protein nesprin-1α (Pare et al., 2005a; Boczek et al., 2019). mAKAP binds >20 different signaling enzymes and gene regulatory proteins, thereby regulating stress-induced gene expression in these excitable cells (Dodge-Kafka et al., 2019). mAKAP was the first AKAP to be shown to be capable of binding an adenylyl cyclase (AC), a phosphodiesterase (PDE), and a cAMP effector, thus having the potential to orchestrate completely compartmentalized cAMP signaling (Dodge et al., 2001; Dodge-Kafka et al., 2005; Kapiloff et al., 2009). By expression of nesprin-1α-localized constitutive active AC and PDE fusion proteins, cAMP at mAKAPα signalosomes has been shown to be sufficient and necessary for neurite extension by embryonic day (E)18 rat hippocampal neurons *in vitro* (Boczek et al., 2019). Inhibition of local cAMP signaling by the PDE-nesprin-1α fusion protein both suppressed forskolin-induced PKA activity detected by a nuclear envelope-localized Forster resonance energy transfer (FRET) PKA reporter (PN-AKAR4) and blocked activity-dependent neurite extension. In contrast, expression of the AC-nesprin-1α fusion protein that increased cAMP levels at mAKAPα signalosomes promoted neurite outgrowth. In addition, anchoring disruptor peptide-mediated displacement of endogenous type 4D3 PDE from mAKAPα signalosomes similarly elevated perinuclear cAMP levels and potentiated neurite extension. mAKAPα signalosomes have been implicated not only in the regulation of neurite extension, but also prosurvival signaling. PDE displacement enhanced retinal ganglion survival *in vivo* after optic nerve crush, consistent with prior findings that the mAKAPα scaffold is required for the neuroprotective effects of exogenous cAMP after crush injury (Wang et al., 2015; Boczek et al., 2019). Using live cell imaging, we now consider the activity-dependent regulation of cAMP levels at mAKAPα signalosomes in neurons, demonstrating local production of cAMP in that compartment and local regulation of neurite extension.

## Materials and Methods

### Plasmids and adenovirus

The cerulean-cpVE172 FRET-based PKA activity sensor AKAR4 was a gift from Jin Zhang, the University of California, San Diego (Depry et al., 2011). To assay PKA activity at the nuclear envelope with spatiotemporal resolution, AKAR4 was expressed in fusion to the N terminus of nesprin-1α (PN-AKAR4). pCAG cyto-RCaMP1h was a gift from Franck Polleux (Addgene plasmid #105014; RRID:Addgene_105014; Hirabayashi et al., 2017). To assay Ca^2+^ at the nuclear envelope using “PN-RCaMP1h,” pS-RCaMP1h-nesprin-1α was constructed by fusing the RcAMP1h cDNA to the 5′ end of a myc-tagged nesprin-1α cDNA. pS-mCherry-Parv-Nesprin and pS-Parv-EGFP-Nesprin plasmids express mCherry-tagged and GFP-tagged *Cyprinus carpio* β-parvalbumin–nesprin-1α fusion proteins, respectively, under the control of the CMV immediate early promoter. pS-mCherry-nesprin and pS-EGFP-nesprin plasmids express nesprin-1α fusion protein controls. New plasmids were constructed by GENEWIZ. Additional details and plasmid sequences will be provided on request.

### Hippocampal neuron isolation and culture

All procedures for animal handling were approved by the Institutional Animal Care and Use Committee at Stanford University. Primary hippocampal neurons were isolated from E18 Sprague Dawley rat embryos of either sex. Briefly, the hippocampal CA1–CA3 region was dissected in PBS medium with 10 mm D-glucose and digested with 0.05% trypsin-EDTA in PBS with 11 mm D-glucose for 30 min at 37°C. The dissociated tissues were centrifuged at 250 × *g* for 2 min and then triturated with fire polished glass pipet in HBSS with calcium and magnesium in plating medium (10% v/v horse serum in DMEM). Dissociated neurons were plated on nitric acid-treated 25 mm cover glass coated with poly-L-lysine in plating medium. Four hours after plating, the medium was replaced with maintenance Neurobasal defined medium supplemented with 1% N2, 2% B27 (Invitrogen), 5 mm D-glucose, 1 mm sodium pyruvate. On days 4–5 in culture, 4 μm arabinosyl cytosine was added to inhibit glial proliferation, and the neurons were plasmid transfected with Lipofectamine 3000 and/or infected with adenovirus.

### Live cell FRET imaging

Imaging was performed using an automated, inverted Zeiss Axio Observer 7 Marianas Microscope equipped with a X-Cite 120LED Boost White Light LED System and a high-resolution Prime Scientific CMOS digital camera. The workstation was controlled by SlideBook imaging and microscope control software (Intelligent Imaging Innovations). The filters used were as follows (Semrock): Dichroics FF459/526/596-Di01 (CFP/YFP/mCherry) and FF409/493/596-Di02 (DAPI/GFP/mCherry); CFP: exciter, FF02-438/24, emitter, FF01-482/25; YFP: exciter, FF01-509/22, emitter, FF01-544/24; mCherry and RcAMP1h: exciter, FF01-578/21, emitter, FF02-641/75; GFP: exciter, FF01-474/27, emitter, FF01-525/45. Cells were washed twice before imaging in PBS with 11 mm D-glucose and perfused during imaging with Tyrode solution (137 mm NaCl, 2.7 mm KCl, 1 mm MgCl_2_, 2 mm CaCl_2_, 0.2 mm Na_2_HPO_4_, 12 mm NaHCO_3_, 11 mm D-glucose, 25 mm HEPES, and 1% BSA) at room temperature (23–25°C) in a perfusion chamber (Warner Instruments). A peristaltic pump (Harvard Apparatus) was used to perfuse the imaging chamber with different drugs in Tyrode solution; delay attributable to perfusion rates was similar across experiments. 100 ms images were acquired every 5–15 s, depending on the tracing. Baseline images were acquired for 2–5 min, with analysis using Slidebooks software. Net AKAR4 FRET for regions of interest was calculated by subtracting bleedthrough for both the donor and acceptor channels after background subtraction. FRET ratio “R” is defined as net FRET ÷ background-subtracted donor signal, with R_0_ being the ratio for time = 0. RCaMP1h intensiometric data are normalized to the intensity (I) at time = 0.

### Neurite extension assays

For neurite extension assays, the cells were cultured for 3 d in maintenance Neurobasal defined medium supplemented with 1% N2, 2% B27 (Invitrogen), and 1 mm sodium pyruvate. On days 3–4 in culture, 4 μm arabinosyl cytosine was added to inhibit glial proliferation, and on day 4, the neurons were co-transfected with pmCherry-C1 and Parv-GFP-nesprin or control GFP-nesprin expression plasmids using Lipofectamine LTX with Plus Reagent (ThermoFisher Scientific, catalog #15338030). KCl (40 mm) was added to the medium after transfection. Two days later, the neurons were fixed and counterstained with Hoechst (Invitrogen, catalog #33342). Images were acquired with a Zeiss 880 confocal microscope by 20× objective tile scan and processed with Fiji ImageJ. The length of the longest neurite for 20–40 neurons per condition was measured for each experiment using ImageJ with the Simple Neurite Tracer plugin.

### Statistical analysis

Statistical analyses were performed using GraphPad Prism. Data are presented as mean ± SEM. Normally distributed datasets by D’Agostino–Pearson omnibus (K2) test were compared by unpaired *t* tests (for two groups) or one-way ANOVA (for three groups) with subsequent Tukey’s *post hoc* testing. Other datasets were analyzed by Mann–Whitney *U* test (for two groups) or Kruskal–Wallis *H* test (for three groups), followed by Dunn’s *post hoc* testing. Repeated symbols are used as follows: single, *p *≤* *0.05; double, *p *≤* *0.01; triple, *p *≤* *0.001.

## Results

### KCl depolarization induces PKA and Ca^2+^ transients at the nuclear envelope

**Figure 1.**
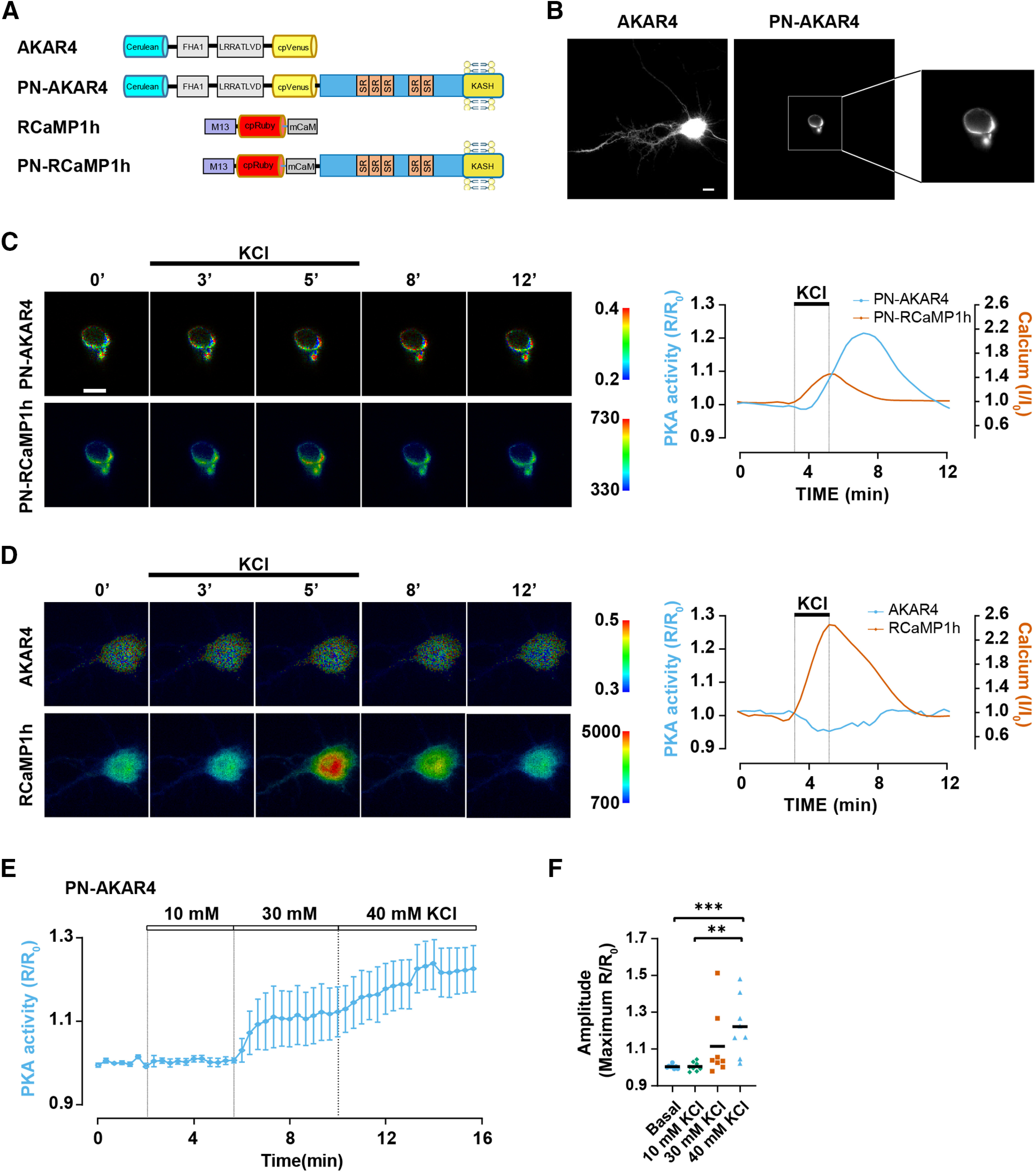
Depolarization selectively activates PKA signaling at the nuclear envelope. ***A***, Sensors used in this study. In AKAR4, phosphorylation of the LRRATLVD peptide by PKA results in FHA1 phospho-peptide binding and increased cerulean-cpVenus FRET (Depry et al., 2011). In RCaMP1h, Ca^2+^ induces the binding of the M13 peptide by the mutant calmodulin domain (mCaM), increasing mRuby fluorescence (Hirabayashi et al., 2017). In the perinuclear-localized sensors PN-AKAR4 and PN-RCaMP1h, nesprin-1α contains five spectrin repeats (SRs) and a transmembrane KASH domain that localizes the protein to the nuclear envelope via binding to SUN domain proteins (Pare et al., 2005a). ***B***, Grayscale CFP images of hippocampal neurons expressing AKAR4 or PN-AKAR4. Scale bar: 10 μm. ***C***, ***D***, Hippocampal neurons were transfected with PN-AKAR4 and PN-RCaMP1h or AKAR4 and RCaMP1h expression plasmids. Representative traces (smoothed with Prism) and pseudocolor images showing FRET (AKAR4) or intensity (RCaMP1h) responses to 40 mm KCl introduced by perfusion. Images were obtained simultaneously for AKAR4 and RCaMP1h and for PN-AKAR4 and PN-CaMP1h. Here and below, a cytosolic region of interest in the soma was measured for the non-localized AKAR4 and RcAMP1h sensors. Scale bar: 10 μm. See Figure 3 for quantification of average responses. ***E***, Averaged trace for PN-AKAR4 response to increasing KCl concentration (10, 30, and 40 mm). Solid line and shaded area indicate mean and SEM, respectively; *n *= 9 from four independent experiments. ***F***, PN-AKAR4 amplitude to different KCl concentrations. Black bars indicate mean values. Datasets were compared by one-way ANOVA and Tukey’s *post hoc* testing; **p ≤ 0.01, ***p ≤ 0.001.

cAMP-dependent PKA signaling is highly compartmentalized in cells by AKAPs that organize localized signalosomes regulating specific cellular processes (Wild and Dell'Acqua, 2018). Given that perinuclear cAMP signaling has been shown to be required for activity-dependent neurite extension (Boczek et al., 2019), we now considered whether KCl-mediated depolarization can regulate cAMP at mAKAPα signalosomes. Cultured primary E18 rat hippocampal neurons were transfected on days 4–5 in culture with expression plasmids for perinuclear-localized PN-AKAR4 or diffusely localized parent AKAR4 PKA activity FRET biosensors (Fig. 1*A*). At the same time, the neurons were co-transfected with plasmids expressing perinuclear-localized PN-RCaMP1h or diffusely localized parent RCaMP1h intensiometric Ca^2+^ sensors (Fig. 1*A*). The neurons were imaged 36–72 h after transfection. As AKAR4 emits cyan and yellow light, while RCaMP1h is red, we were able to image simultaneously similarly localized PKA and Ca^2+^ sensors.

Membrane depolarization with 40 mm KCl for 60 s induced a pronounced increase in perinuclear PKA activity, but had no significant effect on PKA detected with the diffusely localized PKA parent sensor (Fig. 1*B–D*). In addition, whereas 40 mm KCl resulted in robust perinuclear PKA activation, 10 mm KCl did not induce activation of perinuclear PKA, and 30 mm KCl inconsistently resulted in PN-AKAR4 signals (Fig. 1*E*,*F*). This was in contrast to the similarly robust response by AKAR4 and PN-AKAR4 to the transmembrane AC activator forskolin previously observed in these neurons (Boczek et al., 2019). KCl induced Ca^2+^ transients in both compartments, notably with less increase in RcAMP1h signal at the nuclear envelope (Fig. 1*C*,*D*). These results imply KCl depolarization can selectively activate PKA at the nuclear envelope, despite elevating [Ca^2+^] more generally in the cell.

### mAKAPα is required for activity-induced perinuclear PKA signaling

**Figure 2.**
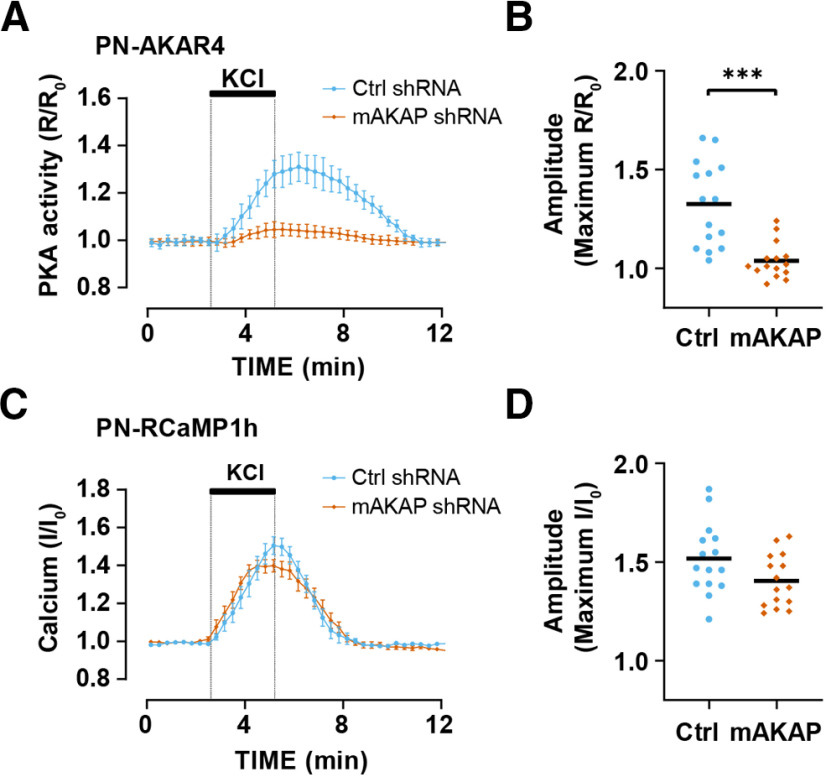
Activity-induced perinuclear PKA activity is mAKAPα dependent. Hippocampal neurons transfected with PN-AKAR4 and PN-RCaMP1h expression plasmids and infected with adenovirus for control or mAKAP shRNA were stimulated with 40 mm KCl; *n *=* *15 for both shRNA and include data from three experiments using separate hippocampal neuron cultures. ***A***, Averaged traces for PN-AKAR4. ***B***, Amplitude of PN-AKAR4 traces. ***C***, Averaged traces for PN-RCaMP1h. ***D***, Amplitude of PN-RCaMP1h traces. Data in ***A***, ***C*** are mean ± SEM; black bars in ***B***, ***D*** indicate mean values. Datasets were normally distributed and compared by unpaired *t* tests; ***p ≤ 0.001.

As PKA is recruited to the nesprin-1α perinuclear compartment by the scaffold mAKAPα, it was likely that KCl-induced PN-AKAR4 signal would be because of activation of mAKAPα-bound PKA. Expression of a shRNA that has been used previously to deplete cells of mAKAP (Pare et al., 2005b; Boczek et al., 2019) inhibited KCl-induced perinuclear PKA activity (Fig. 2*A*,*B*). In contrast, mAKAPα expression was not required for KCl-induced Ca^2+^ transients at the nuclear envelope, such that mAKAP depletion had no effect on Ca^2+^ transients detected with the PN-RCaMP1h sensor (Fig. 2*C*,*D*). Together, these data are consistent with a model in which mAKAPα is required for the recruitment of PKA to the mAKAPα-nesprin-1α perinuclear compartment, but not for the release of Ca^2+^ into that compartment.

### Perinuclear Ca^2+^ dynamics and PKA activity depends on L-type Ca^2+^ channel activity

**Figure 3.**
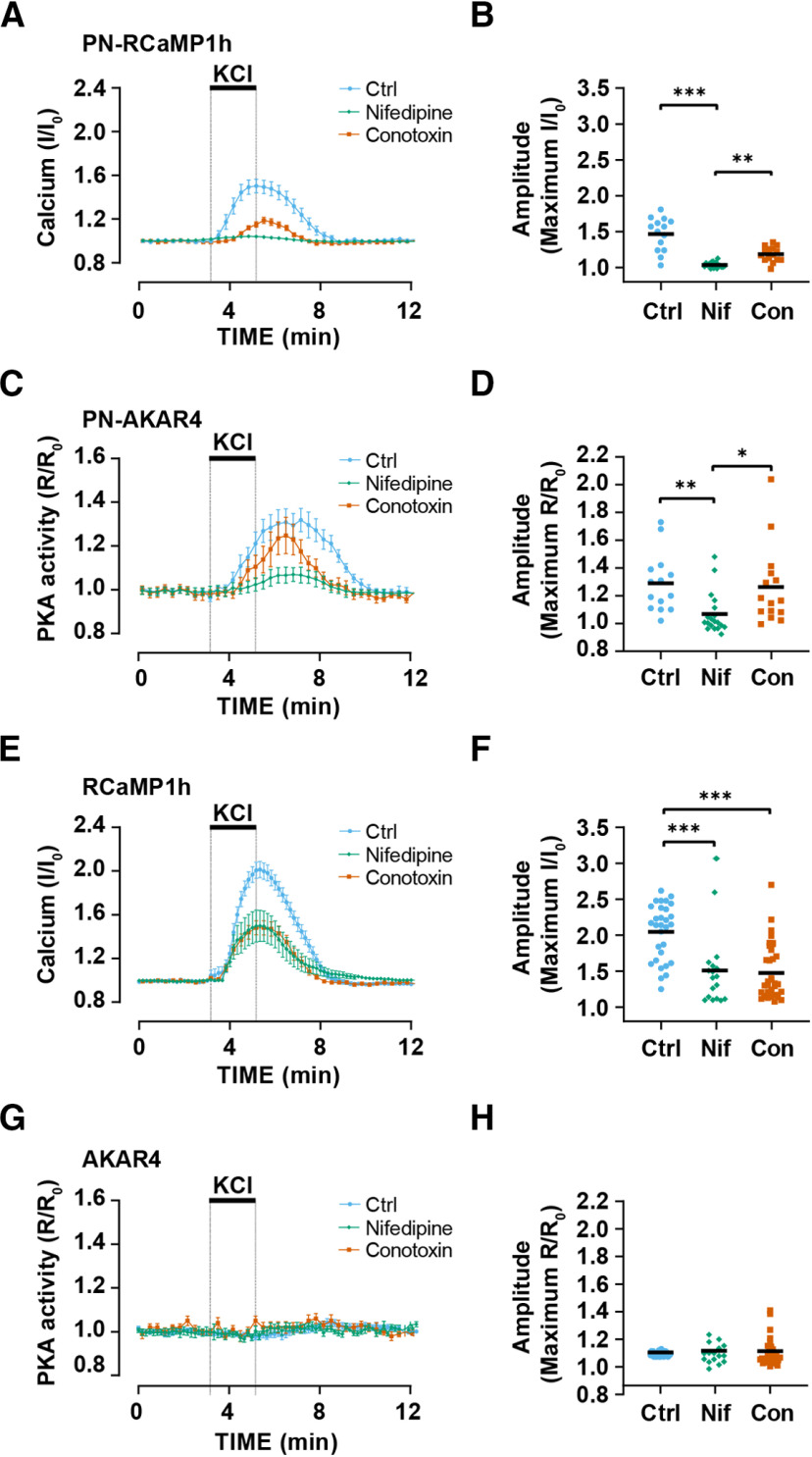
Perinuclear cAMP is regulated by L-type Ca^2+^ channels. Hippocampal neurons expressing PN-AKAR4 and PN-CaMP1h or AKAR4 and RCaMP1h were preincubated with 10 μm nifedipine (Nif; *n *=* *19, 18 for parent and PN-sensors, respectively), 0.5 μm conotoxin GVIA (Con; *n *=* *15, 36), or no inhibitor control (Ctrl; *n *=* *14, 14) before stimulation with 40 mm KCl (bar). ***A***, Averaged traces for PN-RCaMP1h. ***B***, Amplitude of PN-RCaMP1h traces. ***C***, Averaged traces for PN-AKAR4. ***D***, Amplitude of PN-AKAR4 traces. ***E***, Averaged traces for RCaMP1h. ***F*,** Amplitude of RCaMP1h traces. ***G***, Averaged traces for AKAR4. ***H***, Amplitude of AKAR4 traces. Traces show mean ± SEM and are normalized to initial baseline values (R_0_ or I_0_); black bars in ***B***, ***D***, ***F***, ***H*** indicate mean values. Datasets were compared by Kruskal–Wallis and Dunn’s *post hoc* testing; *p ≤ 0.05, **p ≤ 0.01, ***p ≤ 0.001.

We investigated which voltage-gated channels might contribute to Ca^2+^ fluxes in the mAKAPα perinuclear compartment. Preincubation of hippocampal neurons with the L-type Ca^2+^ channel blocker nifedipine inhibited the Ca^2+^ transients detected by the parent diffusely localized RCaMP1h sensor 51% in amplitude and that detected by the PN-RCaMP1h sensor 92% in amplitude (Fig. 3*A*,*B*,*E*,*F*). Preincubation with the N-type Ca^2+^ channel blocker conotoxin GVIA inhibited the Ca^2+^ transients detected by the parent diffusely localized RCaMP1h sensor 55% in amplitude and that detected by the PN-RCaMP1h sensor 60% in amplitude. Accordingly, nifedipine, but not conotoxin GVIA, significantly inhibited KCl-dependent PKA transients detected by the PN-AKAR4-1α sensor (Fig. 3*C*,*D*). Preincubation of the neurons with Ca^2+^ channel blockers did not alter the lack of response of the parent AKAR4 sensor to KCl depolarization (Fig. 3*G*,*H*). Taken together, these results suggest that Ca^2+^-dependent PKA activity in the mAKAPα-nesprin-1α compartment is preferentially dependent on L-type channel activity.

### Chelation of calcium at the nuclear envelope inhibits activity-induced cAMP change

**Figure 4.**
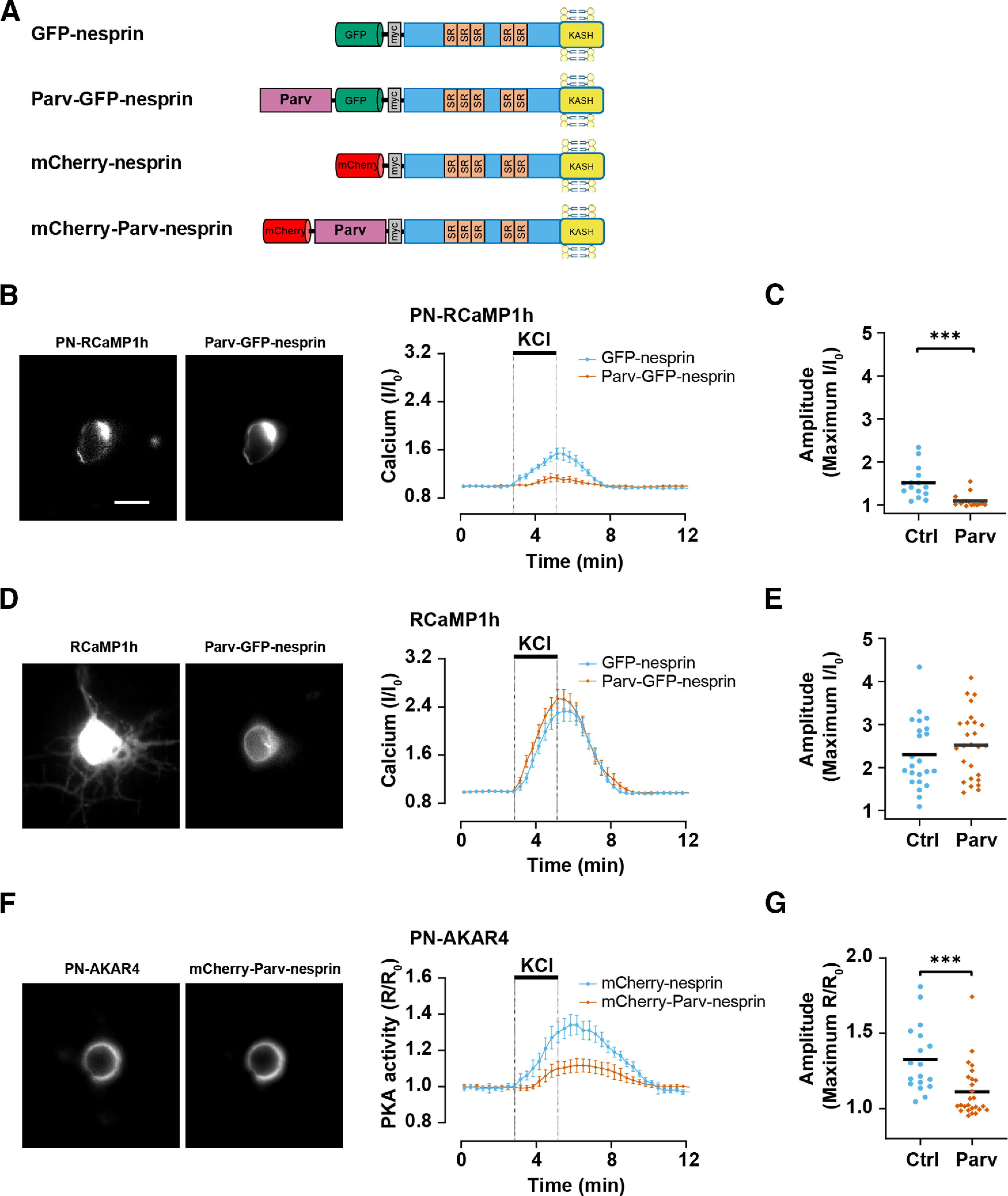
Perinuclear Ca^2+^ is required for cAMP elevation at the nuclear envelope. ***A***, *Cyprinus carpio* β-parvalbumin–nesprin-1α fusion proteins. Nesprin-1α contains five spectrin repeats (SRs) and a transmembrane KASH domain that localizes the protein to the nuclear envelope via binding to SUN domain proteins (Pare et al., 2005a). ***B***, ***C***, Hippocampal neurons expressing PN-RCaMP1h and either Parv-GFP-nesprin (Parv; *n *=* *15) or control GFP-nesprin (Ctrl; *n *=* *15) were stimulated with 40 mm KCl. ***D***, ***E***, Neurons expressing RCaMP1h and either Parv-GFP-nesprin (Parv; *n *=* *26) or control GFP-nesprin (Ctrl; *n *=* *23) were stimulated with 40 mm KCl. ***F***, ***G***, Neurons expressing PN-AKAR4 and either mCherry-Parv-nesprin (Parv; *n *=* *26) or control mCherry-nesprin (Ctrl; *n *=* *18) were stimulated with 40 mm KCl. Traces show mean ± SEM and are normalized to initial baseline values (R_0_ or I_0_); black bars in ***C***, ***E***, ***F*** indicate mean values. Datasets compared by Mann–Whitney *U* test; ***p ≤ 0.001.

As KCl-mediated neuronal depolarization activated perinuclear PKA via an L-type Ca^2+^ channel-dependent mechanism, we next considered whether the Ca^2+^ influx promoting PKA activity was local to mAKAPα signalosomes or elsewhere in the cell besides that compartment. Carp parvalbumin-β is a high affinity (K_a_ = 29 nm) Ca^2+^-binding protein with 10^4^-fold Ca^2+^ selectivity over Mg^2+^ (Wang et al., 2013). To deplete the perinuclear compartment of Ca^2+^, we expressed a parvalbumin-β-nesprin-1α fusion protein tagged with either GFP or mCherry to allow confirmation of intracellular localization (Fig. 4*A*). Co-expression of Parv-GFP-nesprin reduced ∼82% the amplitude of the Ca^2+^ transient induced by depolarization in the perinuclear compartment, but did not affect Ca^2+^ transients detected by the diffusely localized parent RCaMP1h sensor (Fig. 4*B–E*), demonstrating that nesprin-1α localized parvalbumin could only reduce [Ca^2+^] in that compartment. Importantly, expression of the mCherry-Parv-nesprin fusion protein suppressed 66% KCl-induced PKA activity detected by PN-AKAR4 (Fig. 4*F*,*G*), demonstrating that elevation of [Ca^2+^] within the perinuclear compartment is required for full activation of PKA in mAKAPα signalosomes.

### Perinuclear Ca^2+^ is required for activity-dependent neurite extension

**Figure 5.**
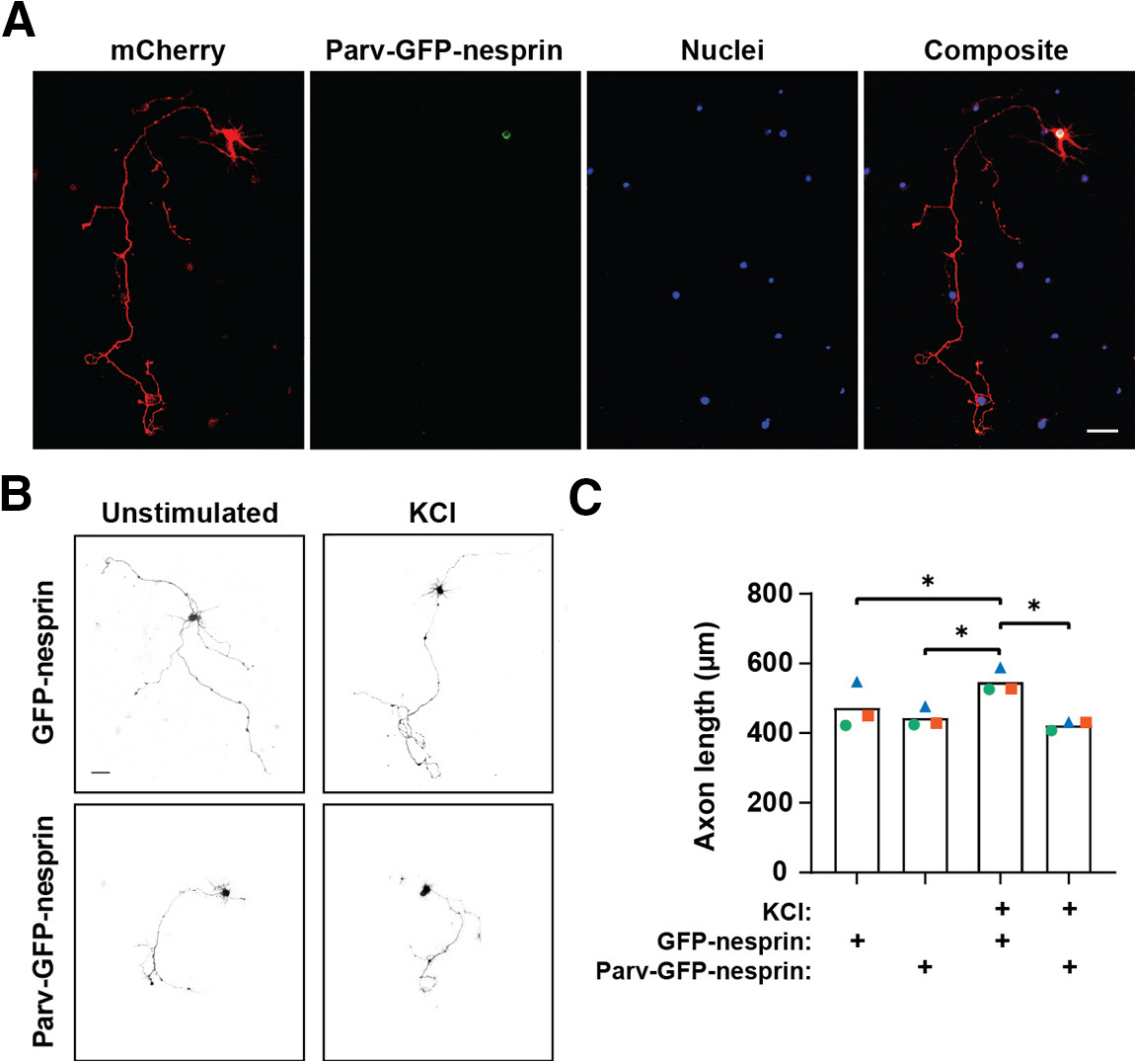
Perinuclear Ca^2+^ regulates neurite extension. ***A***, Images of hippocampal neurons expressing mCherry and Parv-GFP-nesprin-1α and stained with Hoechst nuclear stain in representative neurite extension assay. Scale bar: 50 μm. ***B***, Grayscale images of mCherry fluorescence for hippocampal neurons expressing mCherry and either GFP-nesprin Parv-GFP-nesprin and cultured for 2 d in defined media ±40 mm KCl. Scale bar: 50 μm. ***C***, Means of three independent experiments (differently colored symbols) and average mean (bars) for lengths of the longest neurite are shown; *p ≤ 0.05 as determined by matched two-way ANOVA and Tukey’s *post hoc* testing.

Given that elevated perinuclear [Ca^2+^] was required for activation of mAKAPα-bound PKA, that we previously showed to regulate axon outgrowth (Boczek et al., 2019); we then asked whether selective chelation of perinuclear Ca^2+^ would inhibit axon outgrowth. Hippocampal neurons were transfected with plasmids to co-express either Parv-GFP-nesprin or control GFP-nesprin with mCherry, that served as a whole cell marker (Fig. 5*A*). Measurement of the longest neurite showed that in the absence of KCl, axon length was similar for GFP-nesprin and Parv-GFP-nesprin expressing neurons. KCl stimulation for 2 d induced a 15% increase in axon extension for control GFP-nesprin neurons (Fig. 5*B*,*C*). In contrast, KCl-stimulation induced no increase in axon extension for neurons expressing Parv-GFP-nesprin, demonstrating that perinuclear Ca^2+^ signaling is necessary for activity-enhanced neurite extension.

## Discussion

**Figure 6.**
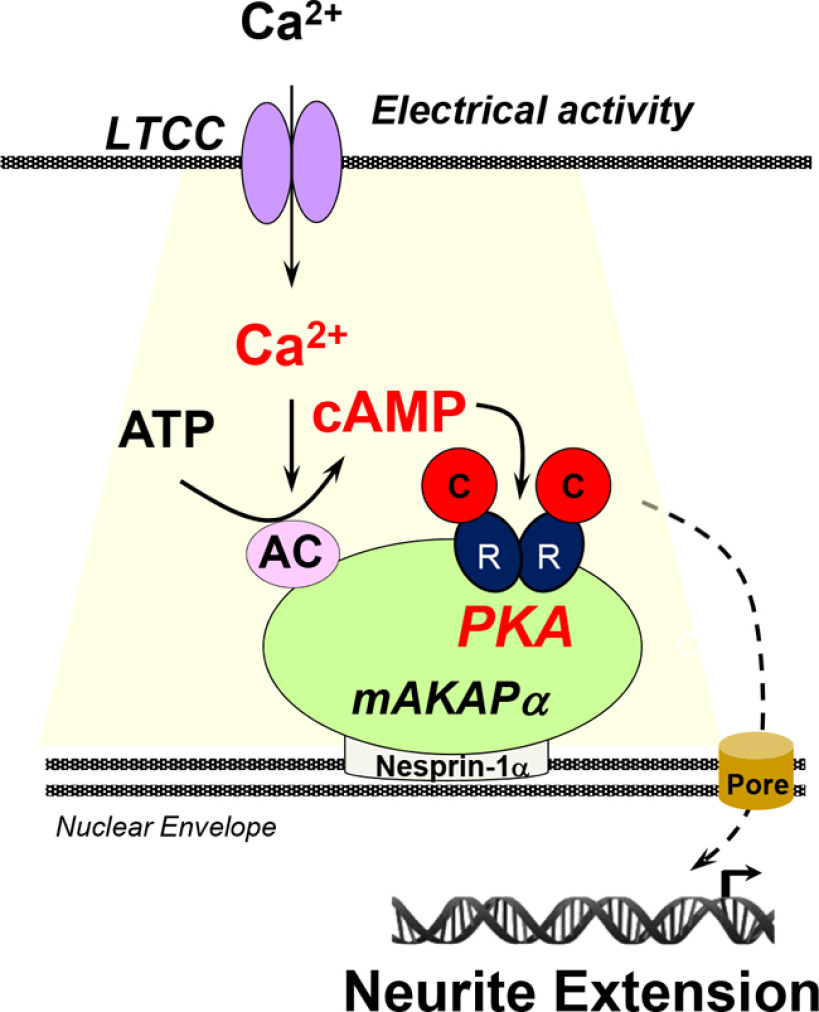
Model of cAMP signaling regulation in the perinuclear compartment. Depolarization of the plasma membrane in hippocampal neurons triggers the opening of L-type Ca^2+^ channels leading to increased perinuclear [Ca^2+^], and activation of Ca^2+^-dependent AC. Perinuclear cAMP binds PKA regulatory (R) subunits, activating PKA catalytic (C) subunits at mAKAPα signalosomes. PKA-phosphorylated effectors that remain to be identified regulate gene expression promoting axon extension. LTCC, L-type Ca^2+^ channels.

Using live cell imaging of primary hippocampal neurons, mAKAPα-bound PKA at the nuclear envelope is shown here to be activated by KCl-mediated depolarization via a mechanism requiring L-type Ca^2+^ channel activity and local increases in [Ca^2+^], promoting neurite extension (Fig. 6). This study extends prior observations regarding mAKAPα signalosomes and neurite extension, including (1) that mAKAPα expression and perinuclear localization is important for neurite extension *in vitro* (Wang et al., 2015; Boczek et al., 2019); (2) that elevated cAMP at mAKAPα signalosomes is sufficient and necessary to induce hippocampal neurite extension *in vitro* (Boczek et al., 2019); and (3) that displacement of the mAKAPα-bound PDE PDE4D3 results in elevated perinuclear cAMP levels and increased neurite extension (Boczek et al., 2019). Surprisingly, KCl-mediated membrane depolarization, which induced neurite outgrowth, increased PKA activity detected with the localized PN-AKAR4 sensor, but not the diffusely expressed parent AKAR4 sensor, despite using a strong KCl stimulus. This was in contrast to prior findings that forskolin activated PKA detected with both sensors (Boczek et al., 2019). Further, relatively high levels (40 mm) of KCl was required for perinuclear PKA activation, consistent with previous findings that mAKAPα-dependent perinuclear signaling is linked to signaling in stressed, but not healthy, neurons (Wang et al., 2015). Neuronal activity, modeled *in vitro* by KCl-mediated depolarization, is a major determinant of CNS neurite extension and neuronal survival, and, moreover, induces these processes via cAMP/PKA-dependent mechanisms (Lipton, 1986; Shen et al., 1999; Goldberg et al., 2002; Corredor et al., 2012). Given additional prior findings regarding the role of mAKAPα signalosomes in retinal ganglion cell survival (Wang et al., 2015; Boczek et al., 2019), we suggest that the data shown herein support a model in which the perinuclear, mAKAPα cAMP compartment is a major node in the intracellular signaling network controlling both axon extension and neuroprotection.

Imaging of neurons expressing a nuclear envelope-localized parvalbumin fusion protein suggests that Ca^2+^ influx induced by KCl depolarization must include elevation of Ca^2+^ at the nuclear envelope in order for mAKAPα-bound PKA to be fully activated. In addition, depletion of Ca^2+^ at the nuclear envelope inhibited KCl-stimulated axon elongation, demonstrating the functional consequence of Ca^2+^ signaling within the perinuclear compartment. Notably, activation of L-type Ca^2+^ channels appears critical for this process, consistent with the previously recognized role of these channels in regulating neuronal gene expression (Wild and Dell'Acqua, 2018). L-type Ca^2+^ channels have also been linked to hippocampal survival signaling in response to iron toxicity (Bostanci and Bagirici, 2013) and have been contrasted with NMDA-mediated Ca^2+^ entry and induction of cell death in hippocampal neurons (Stanika et al., 2012), although neither of these studies examined Ca^2+^ or cAMP signaling at the perinuclear region. Together with our data demonstrating dependence on L-type Ca^2+^ channels for perinuclear Ca^2+^ and cAMP signaling, and previous observations identifying the importance of this compartment for neuronal survival and axon growth (Boczek et al., 2019), these examples support a model in which specific Ca^2+^ signaling pathways converge on mAKAPα at the nuclear envelope to support survival and growth signaling. We cannot exclude that L-type Ca^2+^ channels at sites remote from the nuclear envelope regulate mAKAPα-bound PKA, including dendritic channels important for excitation-transcription coupling (Oliveria et al., 2007). However, our data are consistent with a model in which local influx through L-type Ca^2+^ channels that are near the nucleus confer compartment-specific activation. L-type channels are enriched on the soma of hippocampal neurons (Hell et al., 1993). The Dell'Acqua laboratory has elegantly demonstrated that somatic L-type channels induce nuclear factor of activated T-cells type 3 (NFATc3) transcription factor nuclear translocation via activation of the phosphatase calcineurin (Wild et al., 2019). While NFATc3 translocation in neurons does not appear to be dependent on ryanodine receptors that confer Ca^2+^-induced Ca^2+^ release from intracellular stores (Wild et al., 2019), mAKAPβ in striated myocytes has been shown to bind ryanodine receptors, and L-type channels can induce ryanodine receptor opening and release of stored Ca^2+^ (Marx et al., 2000; Kapiloff et al., 2001; Ruehr et al., 2003). Whether ryanodine receptors that have been detected at neuronal nuclear envelope and can regulate nuclear Ca^2+^ participate in elevating perinuclear Ca^2+^ fluxes at mAKAPα signalosomes will be subject of future studies (Walton et al., 1991; Kumar et al., 2008).

Our findings imply that a Ca^2+^-dependent AC is responsible for local synthesis of cAMP in the mAKAPα compartment (Fig. 6). It is formally possible that Ca^2+^-activates perinuclear cAMP via inhibition of a local cAMP PDE, albeit the PDE that regulates the mAKAPα compartment PDE4D3 is not known to be inhibited by Ca^2+^ signaling. Instead, the binding of an AC by mAKAPα could confer this local regulation. AC1, AC3, and AC8 are activated by Ca^2+^/calmodulin, and AC10 (soluble AC) by Ca^2+^ and bicarbonate (Sadana and Dessauer, 2009). mAKAP has been shown to bind AC5 and AC2, but not AC1 and AC6 when co-expressed in heterologous cells (Kapiloff et al., 2009). Other ACs were not tested for mAKAP binding. mAKAP residues 245–340 binds directly the conserved C1 and C2 catalytic domains of AC5, such that the specificity in mAKAP-AC binding presumably depends on AC sequences not conserved among isoforms. While activating ACs 1–8, forskolin does not activate AC9 and AC10 (Sadana and Dessauer, 2009). As both forskolin and KCl stimulate PN-AKAR4 in hippocampal neurons (as shown here and in Boczek et al., 2019), one might predict that AC3 or AC8 (but not AC1) is responsible for mAKAPα-associated PKA activity. However, forskolin should broadly activate transmembrane ACs in neurons, potentially resulting in a non-specific, non-physiologic diffuse cAMP activation, including in the mAKAPα compartment. AC10 can promote retinal ganglion cell neurite extension and survival *in vitro* (Corredor et al., 2012), and thus AC10 could participate in mAKAPα signalosomes. On the other hand, AC1/AC8 double knock-out did not affect retinal ganglion cell axon growth, but did inhibit the forskolin-potentiated survival of these neurons *in vitro* (Corredor et al., 2012). In addition, other ACs are regulated indirectly by Ca^2+^-dependent protein kinases and phosphatases. Provocatively, while AC1/8 double knock-out reduced KCl-dependent cyclase activity in hippocampal neurons ∼60%, KCl could also activate AC in these cells via activation of calcineurin (Chan et al., 2005). As mAKAPβ binds active calcineurin promoting the dephosphorylation of NFATc and MEF2 transcription factors (Dodge-Kafka et al., 2019), it is possible that KCl and Ca^2+^ activates AC in the mAKAPα compartment via a calcineurin-dependent pathway. The identification of the AC(s) critical for perinuclear cAMP-dependent neurite extension and neuroprotection will require future studies involving specific interference with the expression (RNAi) of individual cyclases and PN-AKAR4 imaging. While it remains to be established in neurons, given the prominent role of mAKAPβ signalosomes in the control of stress-regulated cardiac myocyte gene expression (Dodge-Kafka et al., 2019), cAMP-dependent signaling at mAKAPα signalosomes presumably regulates neuronal gene expression controlling neurite extension. Future studies will be directed at the discovery of mechanisms by which activity-dependent cAMP signaling at mAKAPα signalosomes promote hippocampal neuron neurite outgrowth *in vitro*. Additionally, future studies should explore whether organization of signaling downstream of physiologic (e.g., synaptic) signaling is involved in other phenotypes including homeostatic regulation of activity. Meanwhile, the identification of a cAMP compartment that can promote axon growth and neuroprotection suggests that further study of this compartment is warranted in terms of both basic mechanism and potential translational relevance.

## Synthesis

Reviewing Editor: Douglas Bayliss, University of Virginia School of Medicine Decisions are customarily a result of the Reviewing Editor and the peer reviewers coming together and discussing their recommendations until a consensus is reached. When revisions are invited, a fact-based synthesis statement explaining their decision and outlining what is needed to prepare a revision will be listed below. The following reviewer(s) agreed to reveal their identity: NONE.

Synthesis

This manuscript was seen by two reviewers, who find that the work nicely addresses regulation of cAMP signaling in neurons, providing evidence of a role for L-type Ca channels and mAKAP-dependent perinuclear PKA activation. The major concern with the paper was the lack of any direct examination of the effects of this mechanism on neuronal survival during stress, the ostensible cellular relevance of the work. In addition, you will also note that that the reviewers had recommendations regarding more clear explanations of rationale and methods for data collection, as well as suggestions for image presentation.

Please find the full comments appended below.

Reviewer #1

Advances the Field

Provides new information on regulation of cAMP signalling in neurons. However, manuscript would be substantially strengthened by inclusion of experiments demonstrating that perinuclear PKA activation in cultured hippocampal neurons modualtes neuronal survival. Given they have constructs for manipulating this pathway, such experiments should be relatively straightforward to do.

Comments

In this manuscript the authors have used FRET imaging of a PKA reporter combined with fluorescent calcium imaging to demonstrate local perinuclear activation of PKA in response to KCl-induced depolarisation in cultured primary hippocampal neurons. They further demonstrate that this local activation of PKA is dependent on mAKAPalpha and local Calcium influx through L-type voltage gated calcium channels. The analyses performed are robust and the data generally well presented. The conclusions regarding local signalling in a perinuclear compartment are supported by the data. However, a major weakness of the study is that they do not demonstrate a relationship between their findings and functional changes in neurite behaviour or survival.

Major points:

1. All experiments were performed using neurons stimulated with 40 mM KCl for 60 sec. The authors have selected this strong stimulus because of previous work linking mAKAPalpha perinuclear signalling in stressed but not unstressed neurons. This implies that perinuclear activation of PKA will not occur in neurons stimulated with weaker (non-stressful) stimuli. This needs to be tested. It also will be important to demonstrate if there is a graded or threshold response to different levels of stimulation.

2. Throughout the manuscript the authors argue that their data are relevant to regulation of neuronal survival during stress. However, no data is included demonstrating that manipulation of local (perinuclear) PKA activation impacts on neuronal survival during stress. For example, do the PARV-nesprin constructs which inhibit local calcium and PKA activation alter neuronal response to stress? Inclusion of such experiments would substantially strengthen the manuscript. While the authors cite some studies showing a link between mAPAKalpha and retinal ganglion cell survival following optic nerve crush, this is a completely different cell type and experimental paradigm. The authors need to demonstrate a relationship between perinuclear PKA activation and cell survival in their cell type/system.

Minor points:

1. Some conclusions in the abstract are overstated. For example, on lines 9/10 it is stated that “membrane depolarization by KCl selectively induced activation of perinuclear PKA activity and Ca2+ levels” While the data support selective perinuclear activation of PKA, this is clearly not the case for Ca2+, which is activated throughout the cell (Figure 1D). Indeed, in the text (lines 152-154) the authors state that “KCl depolarisation can selectively activate PKA at the nuclear envelope, despite elevating Ca2+ more generally in the cell”.

Similarly, the statement that “blocking Ca2+ influx through L-type voltage dependent Ca2+ channels preferentially inhibited both perinuclear Ca2+ elevation and PKA activation is rather unclear. A better description comes in the text (lines 174-175), which more accurately reflects the data “ PKA-activity in the mAKAPa-nesprin-1a compartment is more highly dependent upon L-type channel activity”.

2. No data are included supporting the statements at the end of the abstract and in the significance statement regarding enhancement of neuronal survival (see major point 2 above).

3. Images in Figures 1C/D are very small making it difficult to see any detail.

Reviewer #2

Advances the Field

cAMP-dependent signaling has been implicated as a positive regulator of neuronal survival and regeneration. In this study the authors advance these findings and elucidate the underlying mechanisms of how cAMP signaling is regulated.

Comments

In this manuscript the authors provide data to demonstrate that in hippocampus neurons perinuclear cAMP at mAKAPα signalosome compartment is locally regulated by activity dependent Ca<sup>2+</sup> influx. The authors use PKA activity reporter AKAR4 and intensiometric imaging using RCaMP1α fluorescent Ca<sup>2+</sup> sensor to show that membrane depolarization by KCl induces activation of perinuclear PKA activity. Knockdown of mAKAPα specifically inhibited activity-dependent perinuclear PKA activity but not Ca<sup>2+</sup> influx. The authors further show that specifically L-type voltage dependent calcium channels regulate perinluclear Ca<sup>2+</sup> influx and PKA activation.

I have the following specific comments, which should be addressed before this manuscript can be formally accepted for publication;

1) In Fig. 1 the authors use perinuclear or diffused forms of AKAR4 and RCaMP1h to show that KCl mediated depolarization induces PKA and Ca<sup>2+</sup> influx locally at the nuclear envelope. Providing a schematics of these results will help the readers. In Fig. 1D RCaMP1h signal looks all over the nucleus and even intra-nuclear. Are these confocal images? The authors should also test PN-AKAR4 with RCaMP1h in this experiment. Tracing the nucleus particularly in Fig. 1D is difficult. Authors should also provide DIC images with nuclei demarcated.

2) Fig. 2 lacks the rationale behind those experiments and authors should mention the rationale in main text. Authors need to provide representative images and quantification for mAKAP shRNA vs. Control shRNA.

3) Authors should provide representative images in Fig. 4. In Fig. 4B-E, the authors show that co-epression of Parv-GFP-nesprin inhibited Ca<sup>2+</sup> influx induced by KCl, specifically when they used the PN-RCaMP1h but not with the diffused RCaMP1h sensor. I failed to understand this finding as in Fig. 1D the authors show that both these sensors, more so for diffused RCaMP1h, respond to KCl, then why its only the PN-RCaMP1h sensor, which responds to Parv-GFP-nesprin?
